# Management and disease burden of children and adults with severe IgE-mediated food allergy: Are adults the lost population?^☆^

**DOI:** 10.1016/j.waojou.2024.100971

**Published:** 2024-11-21

**Authors:** Katharina Blumchen, Martin Hutter, Sabine Schnadt, Gregor Bushart, Claudia Mailaender

**Affiliations:** a Department of Pediatrics, Division of Pneumology, Allergology, Infectious Diseases and Gastroenterology, Goethe University, Frankfurt, Germany; b DAAB – German Allergy and Asthma Association e.V., Bonn, Germany; c Novartis Pharma GmbH, Nuernberg, Germany

**Keywords:** IgE-mediated food allergy, Risk for anaphylaxis, Health resources, Burden of disease, Children, Adult population

## Abstract

**Background:**

Lacking causal treatment options in most cases, severe IgE-mediated food allergies (IgE-FA) are associated with a high burden of disease due to permanent risk of anaphylactic reactions after accidental allergen ingestion. To date, only few data comparing health resources and burden of disease between the pediatric and adult population are available.

**Objective:**

Our survey aimed to assess the care situation of pediatric and adult patients with severe, self-reported physician-diagnosed IgE-FA.

**Methods:**

The survey was conducted via an online questionnaire consisting of 32 items covering participant demographics, comorbidities, triggers, utilization of health resources, current management and burden of disease of FA, according to age groups (<18 years: proxy report by parents or ≥ 18 years: self-report by adults).

**Results:**

A total of 367 participants (n = 237 children/parents, n = 130 adults) with self-reported physician-diagnosed IgE-FA and physician-prescribed adrenalin autoinjector were enrolled. Emergency training and having an emergency action plan were significantly more common in the pediatric group (81.4%) than in the adult group (36.2%). Children had clearer medical contact points (pediatrician or [pediatric] pulmonologist, 89.0%), while adults visited a variety of specialized physicians according to their FA-related symptoms. Adults were more unsatisfied with their overall coping-strategy for allergen avoidance (18.5%), daily FA management (27.9%), and treating physician (34.4%) than the pediatric group (2.6%/17.0%/14.8%, respectively, p < 0.05).

**Discussion:**

Our data reveal a general undersupply for severe IgE-FA in Germany, with adults being significantly more affected. This may lead to the reported higher burden of disease in this age group. Increasing clearer medical contact points (eg, qualified allergologists specialized in food allergy)—especially for the adult patient population, finding available therapeutic options for this group of patients, and increasing the awareness of severe food allergy in the general population might overcome this problem.

## Introduction

Severe food allergies (FA) are burdensome for the affected individuals and their caregivers with permanent risk of potentially life-threatening anaphylaxis following accidental allergen ingestion.^1^ There are differences such as in prevalence, gender frequency, type of allergens eliciting a reaction, severity of reactions, and quality of life (QoL) for FAs in children compared to adults.

Prevalence data of IgE-mediated food allergy (IgE-FA) are limited.^2^ Self-reports on diagnosis typically show higher prevalence compared to diagnosis by oral food challenge, which is still the gold standard for diagnosing FA.^3^ A recent systemic review and meta-analysis analyzed data published between 2000 and 2021 on FA frequency in Europe.^2^ Authors reported a point prevalence of any self-reported physician-diagnosed FA in the last 12 months of 4.9% (3.8% in children vs. 6.9% in adults). The point prevalence based on positive food challenge was however only 0.8% (0.7% in children vs 1.4% in adults).^2^ Further data suggest FA prevalence in children of 4.2–7.6%^4^^,^^5^ and in adults of 3.7–10.8%,^5^, ^6^, ^7^, ^8^ with higher proportions of male infants and adult females being affected.^5^^,^^7^

Recent data of the European Anaphylaxis Registry reveals that FA-induced anaphylaxis is frequently triggered by ingestion of peanuts, cow milk, hens' egg, and tree nuts, as well as fish, in children,^9^^,^^10^ where cow milk and hens’ egg are prevalent elicitors in the first 2 years, hazelnut and cashew in preschool-aged children, and peanut at all ages in childhood.^11^ In contrast, anaphylaxis in adults is more often caused by wheat, shellfish, hazelnut, soy, and peanuts.^9^^,^^10^

Age also seems to have an influence on the severity of food-induced anaphylaxis (reviewed in Turner et al 2022).^12^ An analysis of fatal anaphylaxis data from the United Kingdom also reported an age-related increase in near-fatal and fatal food-anaphylaxis which persists into mid-adulthood.^13^

Just recently—in February 2024—the United States Food and Drug Administration (FDA) approved omalizumab (Anti-IgE) for IgE-mediated food allergy in adults and children 1 year or older in the United States.^14^ But up to that time there was and still is, such as in Germany, only 1 treatment option with a licensed pharmaceutical product available for food allergic patients. This therapeutic option is an oral immunotherapy only available for children and adolescents with peanut allergy.^15^^,^^16^ Individuals affected by FA other than peanut and being older thus had and still have limited therapeutic options because, for example, in Germany only licensed products can be used in patients. Therefore, consequent avoidance of the specific allergenic food is recommended.^17^ Furthermore, avoidance of allergen traces/unintended allergen presence in very sensitive individuals is still essential for prevention of anaphylactic reactions.^18^ This is not only important at home but in any environment in which food is served (eg, restaurant, holidays, invitations, school/work). Mislabeled ingredients, cross-contact of foods with allergens, and the inclusion of food in daily social activities increase the risk of allergen ingestion. Furthermore, it is not possible to accurately identify individuals most at risk of a severe allergic reaction after accidental ingestion of the offending allergen with the help of, for example, biomarkers.^17^ It is therefore not surprising that adults as well as children with IgE-FA are affected by worries and burden in many aspects of everyday life.^19^^,^^20^ Also, mental health concerns like anxiety about living with food allergy in affected patients and fear for safety and trusting others with the child in caregivers have been reported.^21^ Age seems to have an influence on QoL. A study of pediatric and adolescent patients with FA found that QoL was significantly lower in adolescents compared to children in all areas of daily life (emotional, dietary, and social).^22^

Studies and registries focusing on anaphylactic reactions including food-induced anaphylaxis in children and adults are available.^9^ However, only few data comparing health resources and burden of disease between children and adults with IgE-FA and risk for anaphylaxis have been published to date. Therefore, a survey among pediatric and adult patients with self-reported physician-diagnosed IgE-FA and prescribed adrenalin autoinjector (AAI) was conducted to evaluate the care situation and potential undersupply in the surveyed groups.

## Methods

**Fig. 1:**
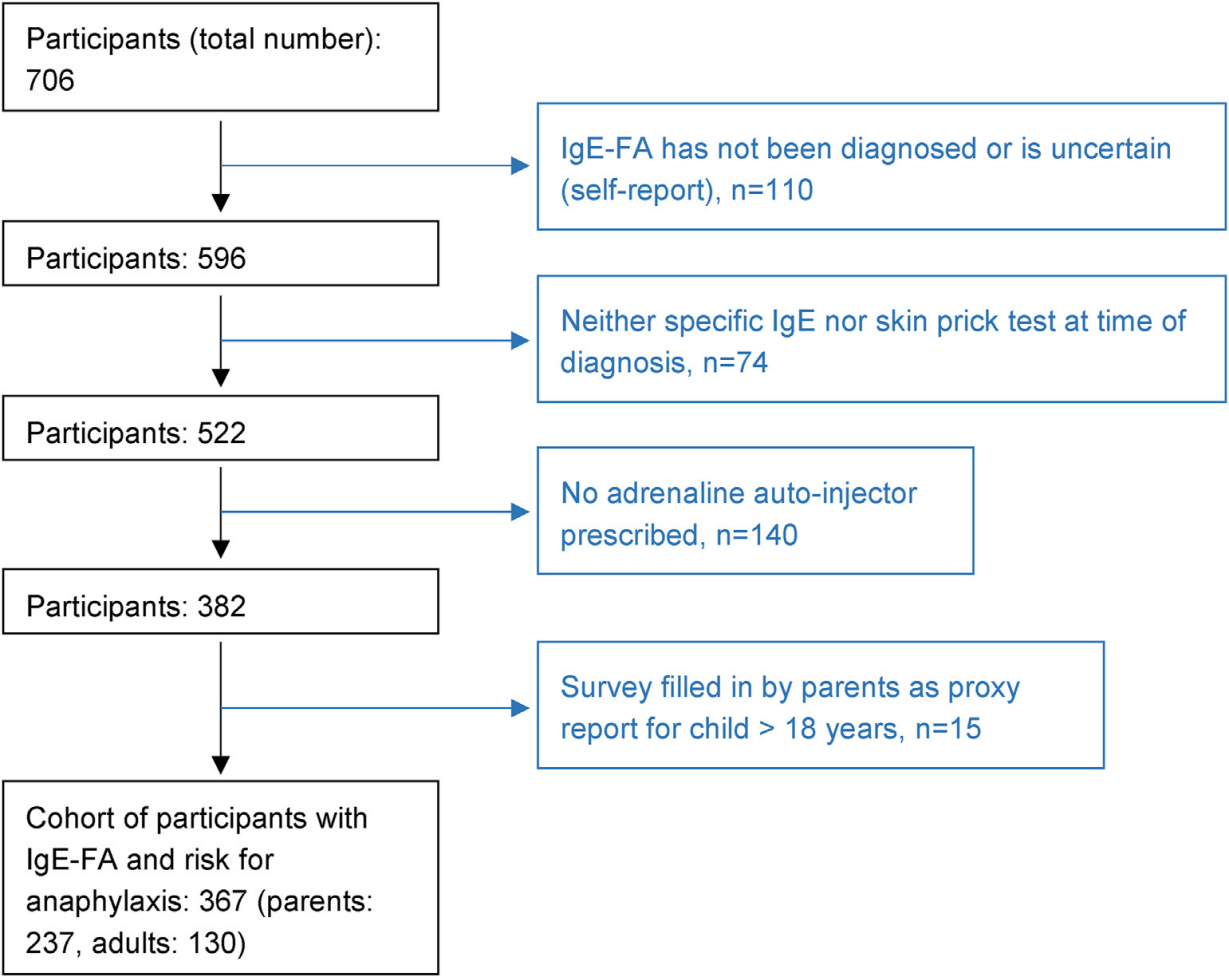
Flow-Chart reflecting the identification of the food allergy cohort with confirmed diagnosis and high risk for anaphylaxis based on the inclusion criteria

The present survey addressing German-speaking food-allergic patients was conducted using a standardized online questionnaire (version 5, December 2021) consisting of 32 questions (see Supplemental Appendix Questionnaire_Food Allergy Survey). In addition to participant demographics, the questions covered the following areas: FA and other comorbidities as well as utilization of health resources for management of the FA in everyday life. Furthermore, participants were asked to report their current situation on management of their/their child's FA and burden of the disease. Inclusion criteria for this data analysis were: 1) self-report of IgE-FA (question within survey: Have you or your child been diagnosed with IgE-mediated food allergy?), 2) self-report on the performance of blood and/or skin prick test at the time of diagnosis (question: How was the diagnosis established [amongst others blood IgE-test, skin prick test]?), and 3) prescription of an AAI due to IgE-FA (question: Which medication have you or your child been prescribed for the FA [amongst others AAI]?). In addition, parents of affected children were asked to complete the questionnaire for their minor children (proxy report), but not for their children ≥18-year-olds (questions: Do you fill in the questionnaire for you or your child? How old are you or your child [related to the person with FA])?). A total of 367 participants fulfilled the cohort criteria relevant to our study (Fig. 1). Participants included both members of the patient organization “German Allergy and Asthma Association” (DAAB) and non-members, with recruitment via Email, Facebook, and Website.

### Data analysis

The analysis of responses and frequencies mentioned was performed according to age groups: <18 years (proxy report completed by parents) or ≥18 years (self-report completed by adults themselves). The p values were calculated using Fisher's exact test as well as Wilcoxon rank-sum test and indicate any differences between groups (proxy report for children vs adults affected). A p value of less than 0.05 was considered as statistically significant.

## Results

### Patient characteristics

The survey was conducted between December 2021 and February 2022, 706 participants answered the survey: 367 participants (130 adults and 237 parents giving a proxy report on their children with IgE-FA) met the inclusion criteria (Fig. 1); the majority (n = 315/344, 91.6%) were members of the DAAB. Overall, approximately half of these food-allergic participants with risk for anaphylaxis/prescription of an AAI were female (Table 1). However, gender differences occurred between the age groups: Among the pediatric food-allergic participants, approximately one-third were female, compared to the adult group where almost all were female (Table 1).

**Table 1:** Patient characteristics of pediatric and adult cohort with severe IgE-FA.

	Proxy report by parents for children with IgE-FA (N = 237)	Adults with IgE-FA (N = 130)	Total (N = 367)	P value
**Gender**				**<0.001**
Male	159 (67.1%)	8 (6.2%)	167 (45.5%)	
Female	78 (32.9%)	122 (93.8%)	200 (54.5%)	
**Age group**				
<6 years	66 (27.8%)		66 (18.0%)	
6−11 years	120 (50.6%)		120 (32.7%)	
12−17 years	51 (21.5%)		51 (13.9%)	
18−35 years		28 (21.5%)	28 (7.6%)	
36−55 years		75 (57.7%)	75 (20.4%)	
≥56 years		27 (20.8%)	27 (7.4%)	
**Allergens (multiple answers possible)**				
Peanut	160 (67.5%)	54 (41.5%)	214 (58.3%)	**<0.001**
Soy	23 (9.7%)	59 (45.4%)	82 (22.3%)	**<0.001**
Lupine	9 (3.8%)	15 (11.5%)	24 (6.5%)	**0.007**
Other legumes	5 (2.1%)	6 (4.6%)	11 (3.0%)	0.207
Tree nut	150 (63.3%)	92 (70.8%)	242 (65.9%)	0.168
Hazelnut	127 (53.6%)	88 (67.7%)	215 (58.6%)	**0.011**
Walnut or pecan	96 (40.5%)	60 (46.2%)	156 (42.5%)	0.321
Cashew or pistachio	90 (38.0%)	31 (23.8%)	121 (33.0%)	**0.007**
Brazil nut or macadamia	49 (20.7%)	38 (29.2%)	87 (23.7%)	0.073
Almond	30 (12.7%)	43 (33.1%)	73 (19.9%)	**<0.001**
Milk	58 (24.5%)	23 (17.7%)	81 (22.1%)	0.149
Egg	93 (39.2%)	24 (18.5%)	117 (31.9%)	**<0.001**
Wheat	23 (9.7%)	29 (22.3%)	52 (14.2%)	**0.002**
Fish	12 (5.1%)	26 (20.0%)	38 (10.4%)	**<0.001**
Crustaceans	10 (4.2%)	34 (26.2%)	44 (12.0%)	**<0.001**
Mollusks	5 (2.1%)	21 (16.2%)	26 (7.1%)	**<0.001**
Sesame	16 (6.8%)	16 (12.3%)	32 (8.7%)	0.083
Mustard	0 (0.0%)	14 (10.8%)	14 (3.8%)	**<0.001**
Celery	5 (2.1%)	55 (42.3%)	60 (16.3%)	**<0.001**
Fruits	35 (14.8%)	84 (64.6%)	119 (32.4%)	**<0.001**
Vegetables	10 (4.2%)	25 (19.2%)	35 (9.5%)	**<0.001**
Meat	2 (0.8%)	4 (3.1%)	6 (1.6%)	0.191
Additives	0 (0.0%)	6 (4.6%)	6 (1.6%)	**0.002**
Spices or herbs	0 (0.0%)	19 (14.6%)	19 (5.2%)	**<0.001**
Other	6 (2.5%)	13 (10.0%)	19 (5.2%)	
**Allergic comorbidities (multiple answers possible)**	196 (87.9%)	123 (98.4%)	319 (91.7%)	**<0.001**
Hay fever/pollen allergy or house dust mite allergy	162 (72.6%)	117 (93.6%)	279 (80.2%)	**<0.001**
Asthma	107 (48.0%)	83 (66.4%)	190 (54.6%)	**0.001**
Eczema/atopic dermatitis	124 (55.6%)	56 (44.8%)	180 (51.7%)	0.058
Hives/Urticaria	17 (7.6%)	33 (26.4%)	50 (14.4%)	**<0.001**
Other	28 (12.6%)	26 (20.8%)	54 (15.5%)	
**Most severe reactions involving (multiple answers possible)**				
Skin or edema	222 (93.7%)	101 (77.7%)	323 (88.0%)	**<0.001**
Eczema	70 (29.5%)	29 (22.3%)	99 (27.0%)	0.142
Eye irritation or upper respiratory tract	155 (65.4%)	95 (73.1%)	250 (68.1%)	0.160
Gastrointestinal	133 (56.1%)	88 (67.7%)	221 (60.2%)	**0.034**
Lower respiratory tract	131 (55.3%)	88 (67.7%)	219 (59.7%)	**0.026**
Cardiovascular	76 (32.1%)	90 (69.2%)	166 (45.2%)	**<0.001**

Significant p values are bold.

The age range was not normally distributed. More than two thirds of the responses were proxy reports by parents answering for their affected children (Table 1). Only 28 of 367 (7.6%) participants were between 18 and 35 years of age (Table 1).

Causal allergens triggering FA differed as expected between the groups (multiple answers were possible and did not especially distinguish between allergens triggering most severe reactions and less severe reactions) (Table 1). Peanut, cashew/pistachio, and egg were significantly more often mentioned as eliciting food allergens in children in comparison to adults. In contrast, adult participants accused significantly more often soy, lupine, hazelnut, almond, wheat, fish, crustaceans, mollusks, fruits, and vegetables as a triggering allergen. In addition, adult participants mentioned mustard, celery, additives, and spices/herbs as triggering allergens with almost no children being affected by these food-allergens.

Among adults, 43.1% (56/130, data not shown) were affected by IgE-FA since childhood. Thus, a significant difference regarding the time since diagnosis was observed between groups: the majority of adults (86/130, 66.2%) suffered from IgE-FA for more than 10 years already, while most of the children (187/228, 82.0%) had the diagnosis since 1−10 years (data not shown).

Most participants (n = 319, 86.9%) suffered from other allergic comorbidities besides food allergy like e.g., hay fever/pollen allergy or house dust mite allergy, asthma, and urticaria (data not shown). As expected, adults were significantly more often affected by these other allergic comorbidities (Table 1). Notably, 72.9% (143/196) of the parents of the FA-allergic children answering for themselves and 52.0% (64/123) of the adults with other allergic comorbidities reported that food allergy is currently the most burdensome condition (p < 0.001, data not shown). Significant differences between children and adults were also observed with respect to their most severe reaction (multiple answers were possible, Table 1). While children more often suffered from skin symptoms, adults reported on significantly higher frequencies of gastrointestinal, lower respiratory tract, and cardiovascular symptoms during their most severe reaction.

### Health resources

The oral food challenge is the gold standard in diagnosis of food allergies but was only performed in less than half of the participants in this survey for verifying diagnosis (Table 2). Significantly more children had undergone an oral food challenge than the adult group (Table 2). Significant differences between the groups were also observed regarding the medical management of FA reflecting an underserved situation for adults (Table 2): Surprisingly, almost three quarters of children (174/237, 73.4%) had received a physician-led training on their emergency medication whereas only 46.2% (60/130) of the adults received this training. Two-fold higher rates in children in comparison to adults were noted for receiving a training/mock pen or an emergency/anaphylaxis action plan by their physician (Table 2). However, both groups reported similarly high rates of food management offers (Table 2): More than 80% of the participants in both groups received information on allergen avoidance by the physician and >50% in both groups reported on having received nutritional counseling in private practices, clinics, external sites or in multi-modal trainings by DAAB including a seminar on how to interpret ingredient-lists of food packages (multiple answers possible). In Germany, it is recommended that parents of children, adolescents as well as adults at risk of anaphylaxis attend a training by AGATE (Working Group Anaphylaxis, Training and Education e. V.) which is a special outpatient educational program focusing on practical aspects of self-management, including role-playing, handling adrenaline auto-injectors, as well as allergen avoidance strategies, behavior in high-risk situations in everyday life, and options of risk management.^23^ Up to now this training program is available for children and to a lesser extend for adults which is reflected in the results of this study (Table 2).

**Table 2:** Utilization of health resources for the management of pediatric and adult participants with IgE-FA.

	Proxy report by parents for children with IgE-FA (N = 237)	Adults with IgE-FA (N = 130)	Total (N = 367)	P value
**Diagnosis by oral food challenge**	120 (50.6%)	39 (30.0%)	159 (43.3%)	**<0.001**
**Management offer**				
Nutritional therapy practice/clinic or external	128 (54.0%)	68 (52.3%)	196 (53.4%)	0.827
**Information**				
Information on allergen avoidance by physician	194 (81.9%)	107 (82.3%)	301 (82.0%)	1.000
Instruction how to use emergency medication (by physician)	174 (73.4%)	60 (46.2%)	234 (63.8%)	**<0.001**
Having received a training pen by physician	134 (56.5%)	35 (26.9%)	169 (46.0%)	**<0.001**
Having received an anaphylaxis-ID/emergency action plan	193 (81.4%)	47 (36.2%)	240 (65.4%)	**<0.001**
**Other training options conducted**				
By German Working Group of Anaphylaxis - Training and Education (AGATE)	96 (40.5%)	10 (7.7%)	106 (28.9%)	**<0.001**
By German Allergy and Asthma Association (DAAB)	125 (52.7%)	55 (42.3%)	180 (49.0%)	0.064
Specialized dietitian/nutritionist	123 (51.9%)	57 (43.8%)	180 (49.0%)	0.156
**Main medical contact person**				
No specific contact person	2 (0.8%)	11 (8.5%)	13 (3.5%)	
Family doctor/general practitioner	10 (4.2%)	34 (26.2%)	44 (12.0%)	
Dermatologist	8 (3.4%)	32 (24.6%)	40 (10.9%)	
ENT doctor (ear, nose, and throat specialist)	3 (1.3%)	12 (9.2%)	15 (4.1%)	
Pediatrician	163 (68.8%)	0 (0.0%)	163 (44.4%)	
Lung specialist/pneumologist (also pediatric pneumologist)	48 (20.3%)	34 (26.2%)	82 (22.3%)	
Other	3 (1.3%)	7 (5.4%)	10 (2.7%)	
**Site of current medical specialist/facility**				**0.002**
Hospital	47 (20.0%)	9 (7.4%)	56 (15.7%)	
Private practice	188 (80.0%)	113 (92.6%)	301 (84.3%)	
**Frequency of physician visits on average**				0.515
≥1 per quarter	41 (17.3%)	31 (23.8%)	72 (19.6%)	
Approx. every 6 months	71 (30.0%)	28 (21.5%)	99 (27.0%)	
1 per year	75 (31.6%)	29 (22.3%)	104 (28.3%)	
<1 per year	50 (21.1%)	42 (32.3%)	92 (25.1%)	

Significant p values are bold.

Within the group of adult participants, a variety of specialized physicians like general practitioners, dermatologists, ENT-doctors (ear, nose, and throat specialist) or lung-specialists were reported to be the main medical contact persons for their FA (Table 2). Interestingly, 8.5% of the adult group (11/130) reported to have no direct medical contact person treating their FA compared to only 2 of 237 (0.8%) in the children group. As expected, children with IgE-FA were mainly treated by a pediatrician or a (pediatric) pulmonologist (combined 211/237, 89.0%). The vast majority of participants was treated by physicians in a private practice (Table 2). However, a significantly higher proportion of children was treated by clinicians within the hospital (Table 2).

Most participants saw their physician at least once per year for their FA, but approx. 20% of all participants (72/367) with no group difference saw their physician ≥1 time per quarter (Table 2).

As expected, adults reported on significantly more episodes of emergency treatments within the past than children. In comparison to 26.3% of the children (54/205), 50.8% of the adults (61/120) reported on ≥3 times of allergic episodes that needed emergency treatment within the past (data not shown). The emergency treatment comprised either the use of an adrenaline autoinjector and/or call for emergency doctor and/or a hospital visit as well as an emergency doctor visit.

### Burden of disease

**Fig. 2:**
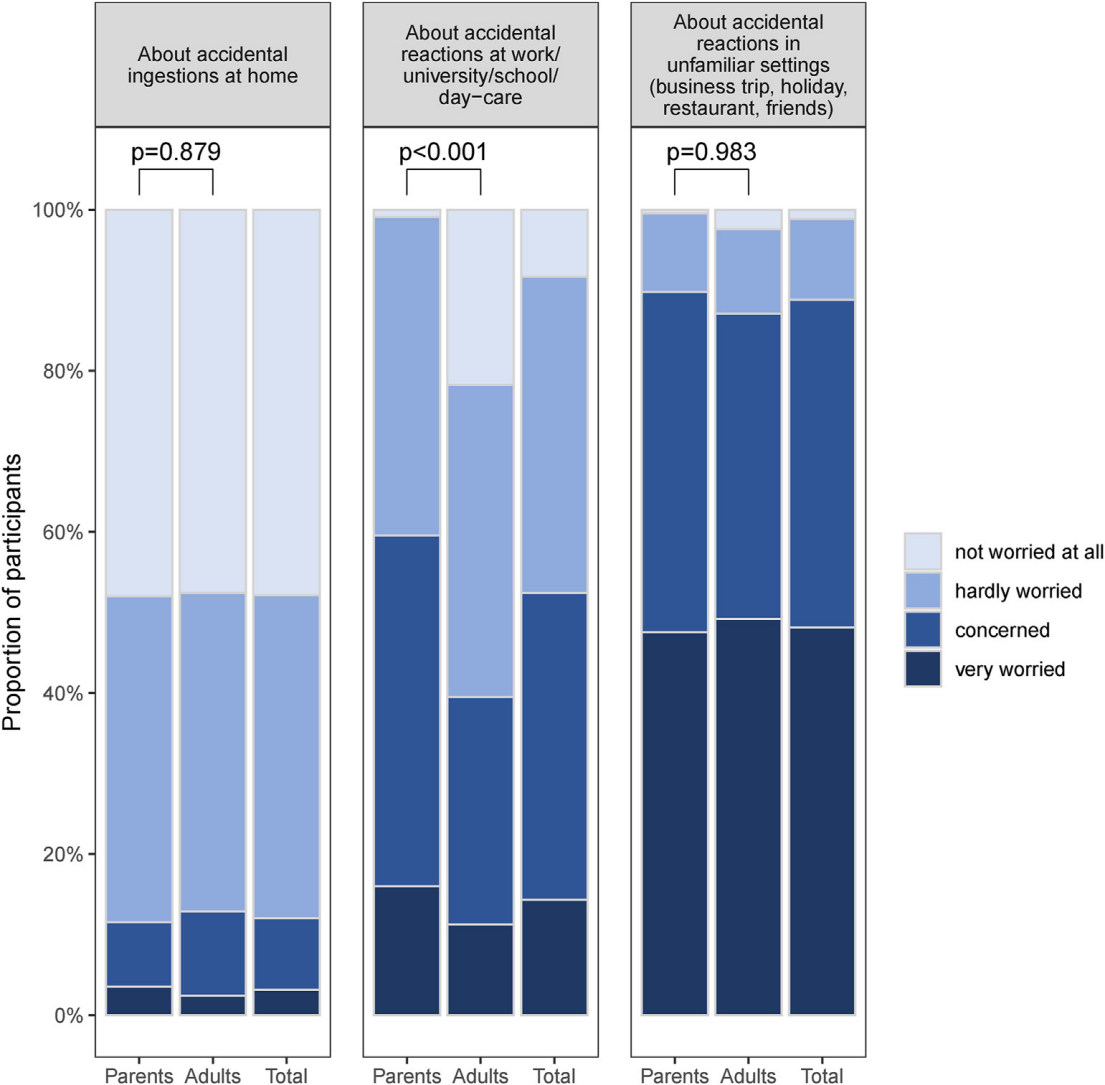
Participants' worries about accidental ingestion of the allergen in different settings (parents n = 237, adults n = 130, total n = 367)

Significant differences between the groups were identified regarding the assessment of the burden of allergen avoidance. Parents gave a self-report on all questions on burden of the disease. While 18.5% of adults (23/124) with severe IgE-FA rated their overall coping-strategy for allergen-avoidance as poor or unsatisfactory, only 2.7% of the parents (6/225) rated this question similar (p < 0.001; total/both groups: 29/349, 8.3%, data not shown), question: “How do you cope with avoidance strategies for your child/for yourself omitting the allergy-causing food(s)?”). No statistically relevant differences between parents and adults resulted from the evaluation of worries about accidental reactions at home or in unfamiliar settings such as business trips, holidays, visits to restaurants or to friends (Fig. 2). However, in both groups worries increased outside the home environment and were strongest in unfamiliar settings/situations. Parents of children with severe IgE-FA reported significantly more worries about accidental reactions at school or day-care than adults at work or university (p < 0.001) (Fig. 2).

**Fig. 3:**
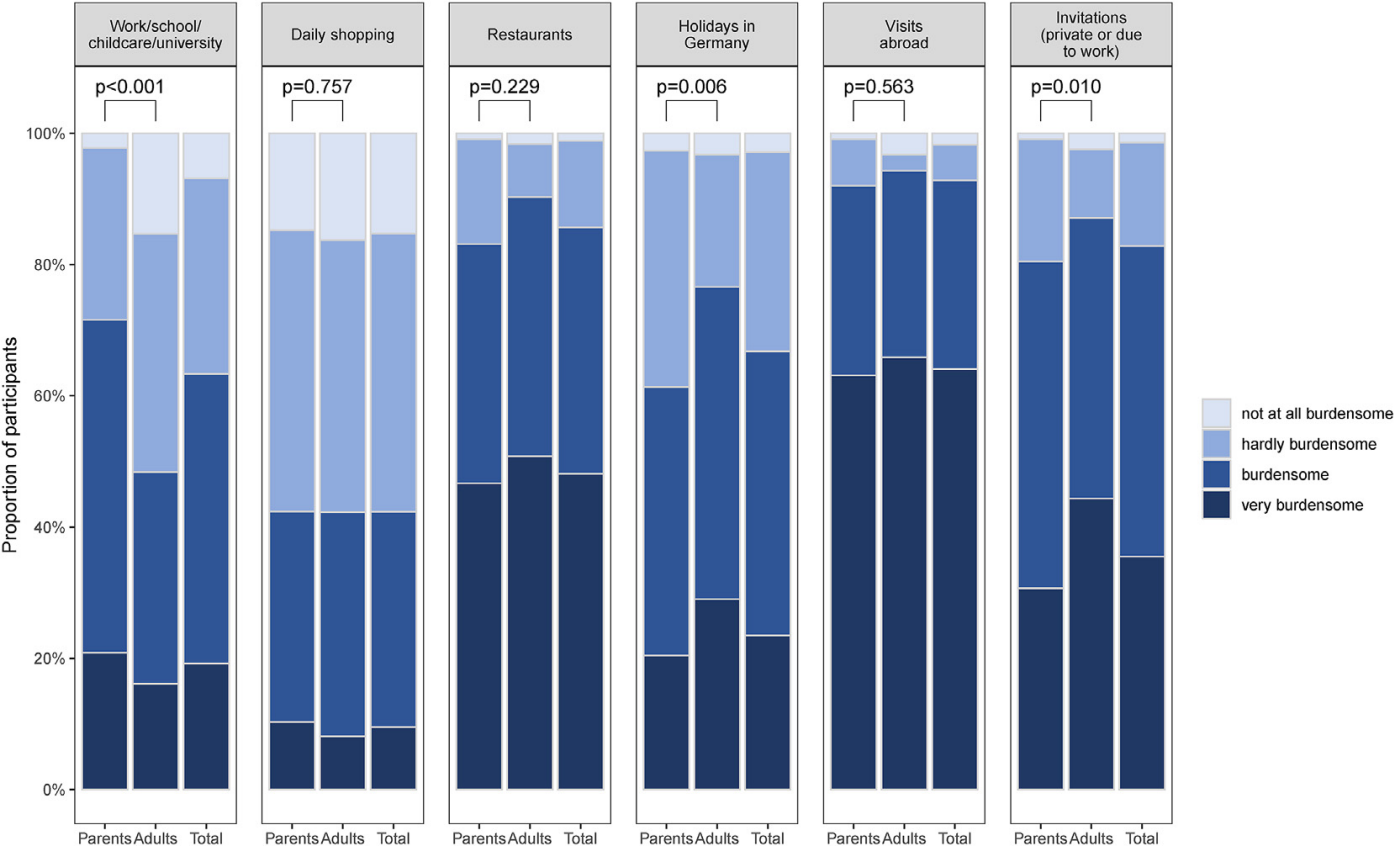
Participants' burden of restrictions due to allergen avoidance in different surroundings (parents n = 237, adults n = 130, total n = 367)

In addition, participants were asked to evaluate the burden of disease concerning restrictions due to allergen avoidance in various specific settings (question: “How burdensome do you perceive your restrictions due to your/your child's food allergy?”, Fig. 3). More than 80% of the participants rated the restrictions due to their own or their child's food allergy as burdensome or very burdensome when visiting a restaurant (299/349, 85.7%), being abroad (323/348, 92.8%), or participating at friends' or work invitations (289/349, 82.8%). Significant differences in burden of disease between parents and adults were identified regarding school/day-care, holidays, and invitations, with higher burden of restrictions in the perception of adults regarding holidays and upon invitations. In contrast, significantly more parents perceived higher burden at school or day-care (Fig. 3). Approximately one-third of the participants (127/349, 36.4%) declared a relevant or high financial burden by the disease with no significant differences between the groups (data not shown).

**Fig. 4:**
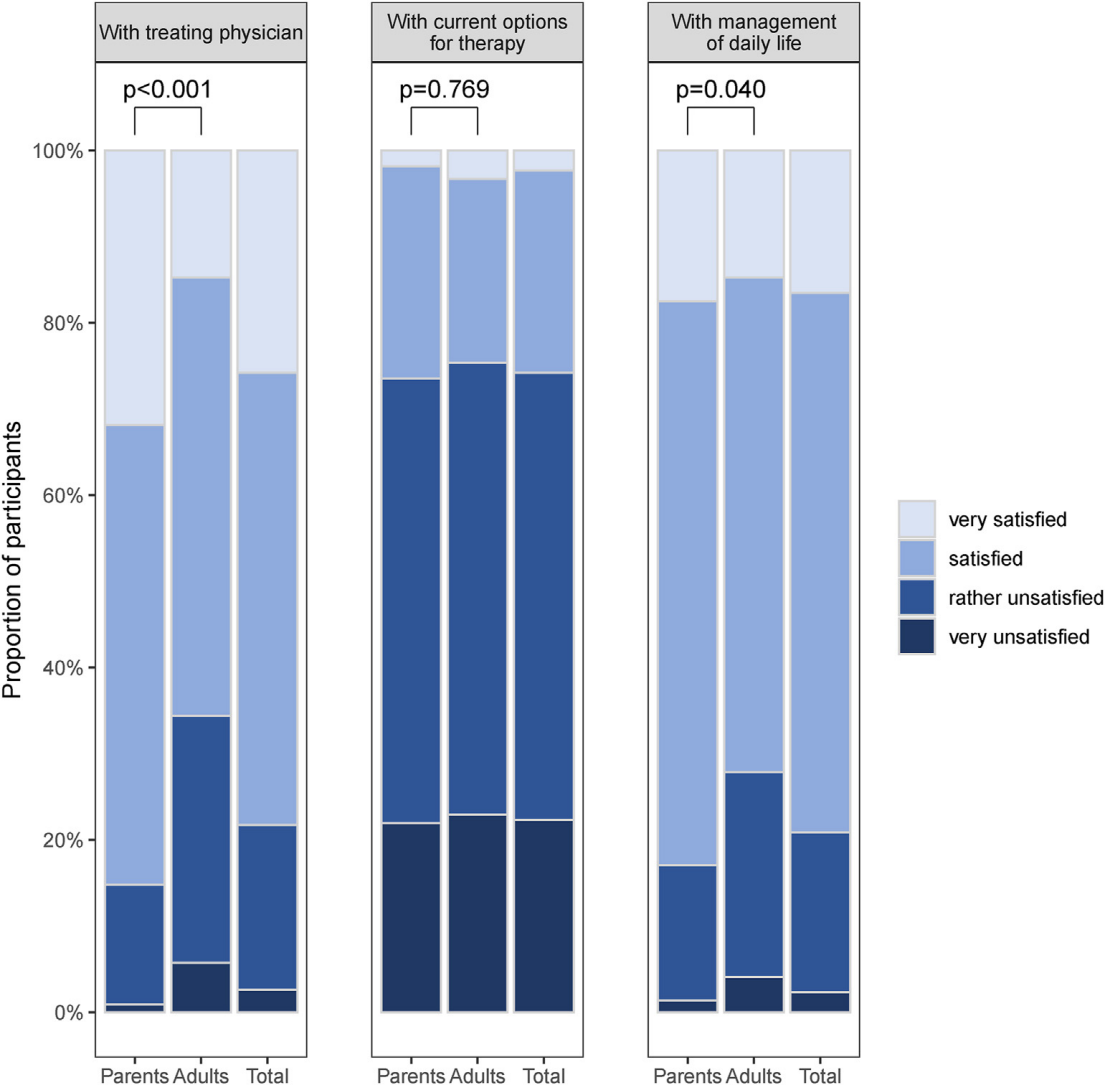
Participants' satisfaction with the treating physician, possible treatment options, and their management of daily life with FA (parents n = 237, adults n = 130, total n = 367)

Finally, participants were asked to report their satisfaction with the treating physician, management of daily life, and therapeutic options (Fig. 4). In comparison to most of the parents (190/223, 85.2%), only 65.6% of adult participants (80/122) were very satisfied/satisfied with their current treating physician. Additionally, a significantly lower proportion of adults (88/122, 72.1%) was very satisfied/satisfied with the day-to-day management of their FA, compared with 83.0% of parents (185/223). Both groups were similarly unsatisfied (very unsatisfied/rather satisfied) with possible treatment options for the disease (parents: 164/223; 73.5%, adults: 92/122; 75.4%) (Fig. 4).

## Discussion

The present survey among pediatric and adult German-speaking patients with self-reported physician-diagnosed IgE-FA and risk of anaphylaxis due to a physician-prescription of an AAI revealed differences in the care situation between these two groups. There is an apparent undersupply of care which especially applies to the adult group which might lead to the reported higher burden of disease in this age group.

Our cohort comprising 367 participants, 130 adults and 237 parents of children, with IgE-FA and risk for anaphylaxis was highly selected. The patient burden in this cohort is also evident from the fact that almost all participants were members of a patient organization. Nevertheless, the cohort is largely coherent with the European Anaphylaxis Registry as well as further literature in terms of allergen spectrum^5^^,^^11^^,^^24^^,^^25^ and different gender distribution between children and adults.^6^^,^^26^ However, the huge proportion of adult females is biased due to more females answering questionnaires, similar to reports, for example, from the US-based survey within the FARE patient registry where 85% of adult patients answering the survey were female.^27^ Another limitation of the cohort is the underrepresentation of the 18–35 age group.

As described above, the allergen spectrum in our survey is largely as expected. Merely, the fact that approximately 42% of adults also indicate peanut as an elicitor for an allergic reaction within this age group is unexpected. The reason could be that more severely affected participants report on peanut as a major allergen.^25^ Higher allergy rates to milk and egg in children with reported anaphylaxis are known.^10^ But in the adult group also 18% indicated milk and 19% egg as a triggering allergen which appears higher than in literature.^10^^,^^24^ Similar to peanut, milk has been reported as a major trigger for fatal or near-fatal anaphylaxis.^25^ Thus milk-allergic adult participants might be overrepresented within the patient organization from which most of the participants were recruited. With regard to the result of triggering allergen, the survey had another limitation not specifying which of the eliciting allergens caused the most severe allergic reaction. Thus, pollen-associated food allergens such as vegetables, fruits, and spices, in most cases not causing anaphylactic reactions, were also mentioned.

A large proportion of the adult participants (43%) reported to be affected since childhood already. Recent data indicate that the IgE-FA-associated burden of disease and impact on QoL are especially high in adults with childhood-onset FA vs adults with later onset of FA.^28^ This is comprehensible as these patients have been exposed to the risk of anaphylaxis due to accidental ingestion over a long period of time. As expected, adults from this study reported on more accidental reactions since 68% of them suffered from diagnosed FA for more than 10 years in comparison to children (18%), thus having more time and opportunities to suffer from accidental reactions. In addition, and possibly influencing QoL, adults reported on significantly more severe symptoms like GI, cardiovascular and lower respiratory symptoms during the most severe reported allergic reaction than parents for their children. Similar published data of the FARE registry report on a higher frequency of anaphylactic reaction in the adult vs the pediatric food-allergic patient group.^27^ Additionally, during food-triggered anaphylaxis cardiovascular involvement is more frequent in adults in comparison to the younger age group.^11^^,^^29^^,^^30^

With regard to utilization of health resources in IgE-FA care, an apparent overall undersupply as well as a dramatic undersupply within the adult group becomes evident. Oral food challenge tests for diagnosis were conducted in only 30% of the adult participants compared to 50% within pediatric group. Only 50% of the pediatric as well as adult group had received a practical nutritional counseling. Additionally, only 46% of the adults received a physician-led training on how to use emergency medication and even less (36%) received an emergency action plan, a trainer pen for the AAI (27%), or AGATE anaphylaxis training (8%) compared to children (73%/81%/57%/41%, respectively). According to current international as well as German guidelines,^1^^,^^31^ all patients with IgE-FA and risk for anaphylaxis should receive a nutritional counseling, written instructions for when and how to use the AAI, training on their specific AAI, also ‒ if possible ‒ an AGATE-training and information on support groups. The underserved situation for adults is further evident by the fact that adults with IgE-FA and risk for anaphylaxis seem to have no specific contact person for their food allergy. In this survey they chose the doctor depending on the symptoms. According to our data, approx. 25% of adult participants each visited a dermatologist, a family doctor, and a pulmonary specialist, respectively, while 9% consulted the ENT. This diversity of type of physicians treating adult food-allergic patients was recently also reported in a survey among German allergologists treating food-allergic patients.^32^ One-third of the adults reported on dissatisfaction with the treating physician and daily disease management.

The above stated underserved situation in adults might be one of the reasons besides others (e.g. different disease perception and past experiences with the disease) for a greater disease burden compared to the better served pediatric population. This is also reflected by our findings regarding higher burden upon holidays and invitations in the adult group. The higher burden in the pediatric cohort with respect to school/day-care might be due to the fact that parents have answered the survey, reflecting their burden of limited control over their children's food intake in such environments. There is also a high proportion of dissatisfaction on the topic of missing therapeutic options in all participants.

The underserved situation for severe food-allergic patients-especially in the adult population-might have multiple reasons: The numbers of qualified allergologists and nursing staff specialized in treating food-allergic adults in Germany might be too low. Physicians might be not qualified enough. Since allergology is a sub-specialization different type of physicians see this patient group; therefore, focused education on food-allergy is more difficult to deliver for a broad spectrum of physicians. Furthermore, time allocated to the patient by adult allergologist might be too short due to health care strains. Future research should closer evaluate the reasons for the underserved situation in the adult patient group. Since our survey was conducted in a German cohort, comparability with other countries regarding different health resources for food-allergic patients is not assured. It would be intriguing to know if other healthcare providers in other countries with e.g. allergology being a separate single discipline where adults and children are seen by the same physician serve the adult population with severe food-allergy in a better way.

A further limitation of this study is that it relied on self-reported data from participants, which may introduce recall bias and subjective interpretation of experiences related to IgE-FA. It also focused on individuals with self-reported physician-diagnosed severe IgE-FA (all participants were prescribed an AAI), excluding those with milder forms and potentially underdiagnosed ones, limiting generalizability of the findings to food allergic patients in general. The relatively small sample size may not fully represent the diversity and complexity of severe IgE-FA, affecting the robustness of the conclusion. Furthermore, the study design did not include a longitudinal follow-up to track changes in management strategies and disease burden over time which could provide a more comprehensive understanding of the long-term challenges faced by individuals with severe IgE-FA. Also, this study did not explore socioeconomic factors that could influence access to healthcare resources.

In conclusion, our results indicate that the different trainings for everyday management (nutritional counseling and emergency trainings) seem to be established in the majority of children with IgE-FA and high risk for anaphylaxis, leading to 83% satisfaction in daily management of the FA. However, the undersupply within the adult group in terms of training offers as well as the general care situation should be urgently improved. This might be achieved by e.g., increasing the awareness of severe food allergy in the general population, increasing the numbers of allergologists treating adults with special interest in food allergy, educating the existing adult allergologists on the topic of severe food allergy, and finding a licensed pharmaceutical therapeutic options for this group of patients.

## Abbreviations

AAI, adrenalin autoinjector; AGATE, Working Group Anaphylaxis, Training and Education e. V.; DAAB, German Allergy and Asthma Association; ENT, Ear nose and throat; FA, food allergy; FDA, Food and Drug Administration; GI, Gastrointestinal; Ig, Immunoglobulin E; QoL, Quality of life.

## Funding

The German Allergy and Asthma Association e.V., Bonn, Germany (DAAB) received a grant from Novartis Pharma GmbH, Germany for data acquisition for carrying out the online patient survey. Novartis Pharma GmbH also provided medical writing support in accordance with Good Publication Practice Guidelines.

## Availability of data and materials

Upon individual request to the corresponding author de-identified data can be made available.

## Authors' contribution to the work

KB: Analysis and interpretation of data, drafting the report and reviewing the written report critically for important intellectual content.

MH: Analysis and interpretation of data, reviewing the written report critically for important intellectual content.

SS: Conception of the study, conducting the study, acquisition of the data, analysis and interpretation of data, reviewing the written report critically for important intellectual content.

GB: Conception of the study, interpretation of data, reviewing the written report critically for important intellectual content.

CM: Conception of the study, interpretation of data, reviewing the written report critically for important intellectual content.

## Ethics approval

According to the local ethics committee approval from an ethic committee was not required since 1.) study data is of untraceable nature, 2.) answering of the survey was voluntary, 3.) the survey was distributed under members of the patient organization “German Allergy and Asthma Association” (DAAB) and non-members, with recruitment via Email, Facebook, and Website, and 4.) the survey was answered by adults only.

## Authors’ consent for publication

All authors gave their final approval of this version of the manuscript to be published. They also gave their agreement to be accountable for all aspects of the work in ensuring that questions related to the accuracy or integrity of any part of the work are appropriately investigated and resolved.

## Declaration of competing interest

KB: Article processing charges for the present manuscript by Novartis Pharma GmbH; Institutional grants by Novartis Pharma GmbH, Aimmune Therapeutics, DBV Technologies, and Hipp GmbH; Consulting fees by Novartis Pharma GmbH, Nestle, Bencard Allergie, Aimmune Therapeutics, ALK, and DBV Technologies; Honoraria by Aimmune Therapeutics, DBV Technologies, Novartis Pharma GmbH, Thermo Fisher Scientific, Viatris Healthcare GmbH, Allergopharma, and Danone; Meeting/travel support by DBV Technologies, Novartis Pharma GmbH, and Aimmune Therapeutics; Participation Data Safety Monitoring Board/Advisory Board by Novartis Pharma GmbH; HM: None; SS: DAAB received a grant for carrying out the online patient survey by Novartis Pharma GmbH; Speakers Honoraria on medical congresses by DBV Technologies; Writing of educational text for website by Aimmune Therapeutics; Registration fee/travel costs to attend congresses by EACCI and DBV; BG and MC: Employees of Novartis Pharma GmbH.
