# Supplementary material for: Management and disease burden of children and adults with severe IgE-mediated food allergy: Are adults the lost population?
Source: World Allergy Organ J. 2024 Nov 21;17(12):100971. doi: 10.1016/j.waojou.2024.100971 (PMC11703708; doi:10.1016/j.waojou.2024.100971)
Supplement: Multimedia component 1 [file mmc1.docx]

***Experiences with food allergies ‒***

***Perspective of patients and parents***

**Demographics:**

1. For whom are you filling in the questionnaire?
   - *For myself*
   - *Form my child*
2. What is the gender of you/ your child (related to the food-allergic person)?
   - *Female*
   - *Male*
   - *Diverse*
3. Have you/ your child been diagnosed with an IgE-mediated food allergy?
   - *Yes*
   - *No*
   - *Not clear*
4. How old are you/ your child (related to the food-allergic person)?

- *< 6 years*
- *6‒11 years*
- *12‒17 years*
- *18‒25 years*
- *26‒35 years*
- *36‒45 years*
- *46‒55 years*
- *56‒65 years*
- *≥ 65 years*

**Food allergy triggers and symptoms:**

1. Which food(s) trigger(s) the allergy(ies)? (multiple answers possible)

- *Peanut*
- *Hazelnut*
- *Walnut / pecan nut*
- *Cashew / pistachio*
- *Brazil nut*
- *Almond*
- *Macadamia nut*
- *Milk*
- *Egg*
- *Wheat and other cereals containing gluten (e.g. rye, barley, other types of wheat such as spelt etc.)*
- *Soy*
- *Fish*
- *Crustaceans (e.g. crab, prawns, shrimps, lobster, crawfish, etc.)*
- *Molluscs (e.g. mussels, squid, octopus, snails etc.)*
- *Sesame seeds*
- *Mustard*
- *Celery*
- *Lupin*
- *Fruit (e.g. kiwi, apple, peach, etc.)*
- *Other, namely: __________________*

1. At which age (in years) did the food-related complaint first occur?

- *< 1 year*
- *1‒2 years*
- *3‒5 years*
- *6‒11 years*
- *12‒17 years*
- *18‒25 years*
- *26‒35 years*
- *36‒45 years*
- *46‒55 years*
- *56‒65 years*
- *≥ 66 years*

1. Which symptoms occurred during the most severe allergic reaction to a food? (multiple answers possible)

- *Skin reaction (redness, itching, wheals, hives)*
- *Eczema exacerbations in atopic eczema*
- *Edema (swelling of the skin, swelling of the mucous membranes)*
- *Eye irritation (itching, redness, conjunctivitis)*
- *Complaints in the upper respiratory tract (runny nose, sneezing, itching in the mouth and throat)*
- *Complaints in the lower respiratory tract (shortness of breath, asthma)*
- *Gastrointestinal complaints (nausea, vomiting, diarrhea)*
- *Cardiovascular complaints (palpitations, drop in blood pressure, fainting, dizziness, etc.)*
- *Other, namely: __________________*

**Diagnosis:**

1. How many months/years ago were you/ your child diagnosed with a food allergy?

- *We are still in the diagnostic phase*
- *In the last 6 months*
- *7‒12 months ago*
- *1‒4 years ago*
- *5‒10 years ago*
- *More than 10 years ago*

1. How was the diagnosis made? (multiple answers possible)

- *Via the medical history/ anamnesis:(relation between consumption and symptoms)*
- *Skin test (skin prick test)*
- *Blood test (detection of IgE)*
- *Provocation test with food (administration of increasing amounts of the food in a clinic)*
- *I cannot remember*
- *None of the above*
- *Other, namely: __________________*

1. Where was the diagnosis made? (if several doctors/persons were involved, please choose the one most relevant to you)

- *Physician in practice (non-hospital doctor)*
- *Hospital Doctor*
- *Other (alternative practitioner, etc.)*

1. Which specialist made the diagnosis? (if several doctors/persons were involved, please choose the one most relevant to you)

- *Family doctor/ general practitioner*
- *Internist*
- *Pediatrician*
- *ENT doctor (ear, nose and throat specialist)*
- *Dermatologist*
- *Lung specialist/pneumologist (also pediatric pulmonologist)*
- *Gastroenterologist (specialist for gastrointestinal diseases)*
- *Other medical specialty, namely: (Please note: "Allergologist" is not a specialist in Germany! Therefore please do NOT enter "Allergologist" here. Please only enter here if a specialist other than one of the above has made the diagnosis) __________________________*

**Medical treatment and care:**

1. How many different doctors (number) have you already visited due to your / your child's food allergy?

- *One*
- *2‒3*
- *4‒5*
- *6‒10*
- *> 10*

1. Which (therapy) offers have you / your child received from your / their doctor so far? (multiple answers possible)

- *Information on avoiding the allergy trigger from the doctor*
- *Nutritional therapy in the practice / hospital*
- *Referral to an external nutritional therapist*
- *Reference to the DAAB (German Allergy and Asthma Association)*
- *Handing out of an allergy passport*
- *Prescribing medication*
- *Training on the use of the emergency kit by the doctor*
- *Training pen for adrenaline auto-injector was handed out by the doctor*
- *Handing out of an anaphylaxis passport*
- *Handing out of an anaphylaxis emergency plan*
- *Other, namely: __________________*

1. Which medication(s) have you / your child been prescribed due to the food allergy? (multiple answers possible)

- *Antihistamine (liquide, drops, tablets, e.g. Fenestil^©^, Ceterizin etc.)*
- *Cortisone (liquide, tablets, suppositories, e.g. Okrido^©^, Celestamine^©^ etc.)*
- *Adrenaline autoinjector (e.g. Fastjekt^©^, Jext^©^, Emerade^©^, Anapen^©^)*
- *Asthma emergency spray (e.g. salbutamol, ipratropium)*
- *Other*
- *None*

1. Which specialist is currently your main contact for the treatment of your / your child's food allergy? (Please check only one as your main contact)

- *Family doctor/ general practitioner*
- *Internist*
- *Pediatrician*
- *ENT doctor (ear, nose and throat specialist)*
- *Dermatologist*
- *Pulmonologist (also pediatric pulmonologist)*
- *Gastroenterologist (specialist for gastrointestinal diseases)*
- *Other medical specialty, namely: (Please note: "Allergologist" is not a specialist in Germany! Therefore please do NOT enter "Allergologist" here. Please only enter here if a specialist other than one of the above has made the diagnosis) __________________________*
- *I have no contact person*

1. The doctor named in question 15 works in a

- *practice*
- *hospital*

1. On average, how often do you visit a doctor due to your / your child's food allergy?

- *2‒3 per quarter*
- *Once per quarter*
- *Approx. every 6 months*
- *Once a year*
- *Less than once a year*

**Further education and information:**

1. Where else do you get information about your / your child's food allergy, outside the doctor's office? (multiple answers possible)
   - *Not at all*
   - *Patient organization*
   - *Support group*
   - *Internet (blogs, social media)*
   - *Newspaper, magazines (daily and general press)*
   - *Books, patient support guides*
   - *Specialist literature (medical journals, specialist books)*
   - *Other, namely: __________________*
2. Which other training opportunities ‒ outside the doctor's practice ‒ have you /your child taken advantage of so far? (multiple answers possible)
   - *AGATE anaphylaxis training*
   - *DAAB online seminars*
   - *Advice from allergy-trained nutritionists*
   - *Other, namely: __________________*

**Emergency treatment:**

1. How often have you / your child had to receive emergency treatment for food allergies? (This includes the use of an adrenaline auto-injector, emergency visit to a doctor or hospital, emergency medical treatment)

- *> 5 times*
- *3‒5 times*
- *1‒2 times*
- *Never*

1. How and how often was emergency treatment required? (multiple answers possible)

|  | *Never* | *Once* | *2-3 times* | *3-4 times* | *4-5 times* | *> 5 times* |
| --- | --- | --- | --- | --- | --- | --- |
| *Autonomous use of the emergency kit without consulting a doctor / hospital* |  |  |  |  |  |  |
| *Autonomous use of the adrenaline autoinjector with subsequent visit to the doctor / hospital* |  |  |  |  |  |  |
| *At the doctor's practice (e.g. family doctor / pediatrician)* |  |  |  |  |  |  |
| *Emergency room / outpatient treatment in a hospital with own arrival* |  |  |  |  |  |  |
| *Via emergency call and admission to hospital* |  |  |  |  |  |  |
| *Other:* |  | | | | | |

1. How satisfied were you with the emergency medical care for the most severe anaphylactic reaction?

*1 (very unsatisfied) 10 (very satisfied)* ***_________________________________________________________***

**Other diseases:**

1. Do you / your child currently have any other chronic allergic diseases? (multiple answers possible)
   - *Don't know*
   - *Yes, asthma*
   - *Yes, hay fever / pollen allergy*
   - *Yes, house dust mite allergy*
   - *Yes, eczema, atopic dermatitis*
   - *Yes, hives of a different cause than the food*
   - *Yes, nasal polyps*
   - *Other, namely: __________________*
   - *No*
2. Which of your / your child's allergic diseases is currently bothering you the most?
   - *Food allergy*
   - *Asthma*
   - *Hay fever / pollen allergy*
   - *House dust mite allergy*
   - *Eczema / atopic dermatitis*
   - *Hives of other cause than the food*
   - *Nasal polyps*
   - *Other, namely: __________________*

**Food allergy in daily life:**

How well do you manage with your own / your child's avoidance strategy (omitting the allergy-causing food[s])?

- - *1 very good*
  - *2 good*
  - *3 satisfactory*
  - *4 sufficient*
  - *5 poor*
  - *6 unsatisfactory / very poor*

1. How concerned are you that you / your child may have unintentional contact with the allergenic food(s)? (Please give one answer per line)

|  | *Not worried at all* | *Hardly worried* | *Concerned* | *Very worried* |
| --- | --- | --- | --- | --- |
| *At home* |  |  |  |  |
| *At work / day-care/ school / university* |  |  |  |  |
| *In unfamiliar settings (business trip, holiday, restaurant, with friends)* |  |  |  |  |

1. How stressful do you find the restrictions caused by your / your child's food allergy? (even if a scenario such as a stay abroad does not correspond to your current life situation, please try to imagine it and answer accordingly) Please give one answer per line.

|  | *Not at all burdensome* | *Hardly burdensome* | *Burdensome* | *Very burdensome* |
| --- | --- | --- | --- | --- |
| *Workplace / daycare / school / university* |  |  |  |  |
| *Daily shopping* |  |  |  |  |
| *Restaurant* |  |  |  |  |
| *Holidays in Germany* |  |  |  |  |
| *Visits abroad* |  |  |  |  |
| *Invitations (due to work, private)* |  |  |  |  |

1. How severe do you feel the financial burden caused by your / your child's food allergy?

- No financial burden at all
- Hardly any / moderate financial burden
- Noticeable / significant financial burden
- High financial burden

**Current situation and wishes in regards to the food allergy:**

1. How satisfied are you currently with ...

(Please give one answer per line)

|  | *Very satisfied* | *Satisfied* | *Rather unsatisfied* | *Very unsatisfied* |
| --- | --- | --- | --- | --- |
| *... the medical care, i.e. how well do you/your child feel cared for by the doctor currently treating you?* |  |  |  |  |
| *... the current therapeutic options for food allergies?* |  |  |  |  |
| *... your options of allergen avoidance?* |  |  |  |  |
| *... your everyday food allergy management?* |  |  |  |  |

1. What would be your greatest wish in connection with your / your child's food allergy?

*___________________________________________________________________________*

1. What would you like to achieve with a therapy for your / your child's food allergy?

*___________________________________________________________________________*

1. Are you a member of the DAAB?

- Yes
- No
